# Deep inspiration breath‐hold technique guided by an opto‐electronic system for extracranial stereotactic treatments

**DOI:** 10.1120/jacmp.v14i4.4087

**Published:** 2013-07-08

**Authors:** Cristina Garibaldi, Gianpiero Catalano, Guido Baroni, Barbara Tagaste, Marco Riboldi, Maria Francesca Spadea, Mario Ciocca, Raffaella Cambria, Flavia Serafini, Roberto Orecchia

**Affiliations:** ^1^ Medical Physics Unit European Institute of Oncology Milano Italy; ^2^ Radiation Oncology Unit MultiMedica Clinical Institute, Sesto S. Giovanni Milano Italy; ^3^ Bioengineering Division Dipartimento di Elettronica, Informazione e Bioingegneria Politecnico di Milano Milano Italy; ^4^ Bioengineering Unit Centro Nazionale di Adroterapia Oncologica Pavia Italy; ^5^ Department of Experimental and Clinical Medicine Magna Graecia University Catanzaro Italy; ^6^ Medical Physics Unit Centro Nazionale di Adroterapia Oncologica Pavia Italy; ^7^ Department of Radiation Oncology Sant'Anna Hospital Como Italy; ^8^ Department of Radiation Oncology European Institute of Oncology Milano Italy; ^9^ Department of Health Sciences Università degli Studi di Milano Milano Italy

**Keywords:** deep inspiration breath‐hold, extracranial stereotactic treatment, optoelectronic system, organ motion

## Abstract

The purpose of this work was to evaluate the intrapatient tumor position reproducibility in a deep inspiration breath‐hold (DIBH) technique based on two infrared optical tracking systems, ExacTrac and ELITETM, in stereotactic treatment of lung and liver lesions. After a feasibility study, the technique was applied to 15 patients. Each patient, provided with a real‐time visual feedback of external optical marker displacements, underwent a full DIBH, a free‐breathing (FB), and three consecutive DIBH CT‐scans centered on the lesion to evaluate the tumor position reproducibility. The mean reproducibility of tumor position during repeated DIBH was 0.5±0.3mm in laterolateral (LL), 1.0±0.9mm in anteroposterior (AP), and 1.4±0.9mm in craniocaudal (CC) direction for lung lesions, and 1.0±0.6mm in LL, 1.1±0.5mm in AP, and 1.2±0.4mm in CC direction for liver lesions. Intra‐and interbreath‐hold reproducibility during treatment, as determined by optical markers displacements, was below 1 mm and 3 mm, respectively, in all directions for all patients. Optically‐guided DIBH technique provides a simple noninvasive method to minimize breathing motion for collaborative patients. For each patient, it is important to ensure that the tumor position is reproducible with respect to the external markers configuration.

PACS numbers: 87.53.Ly, 87.55.km

## I. INTRODUCTION

Tumors in the thorax and upper abdomen are subject to respiratory‐driven motion. In conventional radiotherapy, large safety margins up to 2.0 cm are added to the clinical target volume (CTV) to account for breathing motion and setup errors.^(1)^ Respiration motion also causes artifacts during image acquisition, such as warping of the target volume and erroneous positional and volumetric information.^(2)^

There are three techniques, clinically implemented, able to reduce the effects of respiratory motion. The first is based on abdominal pressure obtained with a plastic plate, often applied to extracranial stereotactic treatments using a body frame.^(3)^ The second technique involves the irradiation delivery at a predefined phase of the respiratory cycle, by gating the accelerator while the patient is freely breathing. Methods for gating include monitoring motion of the abdominal wall, spirometer, laser sensor system, and marker tracking.^4^, ^5^, ^6^, ^7^ A disadvantage of gating is decreased delivery efficiency, with longer imaging and treatment times. The third technique requires the patient to perform specific maneuvers of respiration, either voluntary^8^, ^9^, ^10^ or with the aid of devices.^11^, ^12^, ^13^, ^14^, ^15^ Stock et al.^(16)^ developed a noninvasive method based on the opto‐electronic tracking of passive markers. The breath‐hold technique can be performed either at end of expiration, more reproducible but less comfortable for the patient, or at end of inspiration.^(17)^ Deep inspiration breath‐hold has particular advantages in lung tumors, allowing a reduction of lung density with a decrease of normal tissue in the high‐dose region. This has the potential for dose escalation for the same calculated lung morbidity, even without margin reduction. In order to avoid severe pulmonary toxicity, lung volume receiving more than 20 Gy should not exceed 20% of the total volume.^(18)^ Barnes et al.^(19)^ showed a 32.5% reduction of the lung volume receiving more than 20 Gy using a DIBH technique. Furthermore, the reduced target motion during DIBH may justify a smaller safety margin from CTV to planning target volume (PTV).^(20)^

Nevertheless, in some cases the distance of the PTV to the OARs may increase for other breathing phases and therefore warrant a different phase than DIBH.

In this work, we investigated the use of DIBH technique in stereotactic treatments of lung and liver lesions using two infrared optical tracking systems, ExacTrac (BrainLAB AG, Germany) and ELITE (BTS S.p.a., Milan, Italy), in the attempt to either increase the dose or reduce the safety margin. To guide the DIBH we used the ELITE system, which has already been used in our Institute to evaluate setup errors and their dosimetric consequences in breast treatments,^21^, ^22^ as well as in frameless body stereotactic treatments.^23^, ^24^

We first performed a feasibility study on three voluntary subjects to evaluate the reproducibility of the technique, followed by an analysis on eight patients to correlate the patient surface and the tumor position reproducibility. Finally, the results of the first 15 patients treated with the DIBH protocol have been reported.

## II. MATERIALS AND METHODS

### A. Infra‐red optical tracking

Both optical tracking systems, ExacTrac and ELITETM, use two infrared cameras, which are mounted in the ceiling of the linac vault, to track passive optical markers (radius: 5 mm) placed on selected skin landmarks. A calibration procedure is used in order to determine the position of any passive optical marker relative to the isocenter. Both systems were calibrated with respect to a common isocentric reference frame and they provided submillimetric accuracy in markers 3D localization.^(25)^

The optical markers were attached to the patient by using an adhesive backing before CT scanning, and their position was referenced with ink marking to aid reapplication for each treatment fraction. CT scan data were sent to the treatment planning system (TPS) (BrainScan, BrainLAB AG), where the isocenter was established relative to the stereotactic coordinate system defined by the optical markers configuration which was used for positioning.

ExacTrac was the reference system to set up the patient in free breathing, providing the operator with the corrective translations and rotations. ELITE was the reference system for guiding the patient during the DIBH in both CT and treatment rooms. A specific software was implemented to provide the operators with the real‐time 3D displacements of the optical markers configuration with respect to the reference one.

### B. Feasibility study of the DIBH technique

#### B.1 Volunteer study

In order to assess the reproducibility of the technique as a function of the specific feedback to the patient, a feasibility study was performed on three voluntary subjects. Five consecutive acquisitions of 3D marker positions were performed for each subject lying in supine position on the CT couch during a 15 sec DIBH with two feedback conditions: i) verbal instructions by an operator, based on real‐time feedback of marker displacements; ii) direct visual feedback to the subject.

#### B.2 CT patient study

A CT study was performed on eight patients. Only collaborative patients with a Karnofsky index >70 were enrolled in the feasibility study, as well as in the treatment protocol. The patient characteristics are shown in Table 1 (patients #1‐7, #16). Patients were immobilized supine in an individualized vacuum cushion with an arm holder.

**Figure 1:**
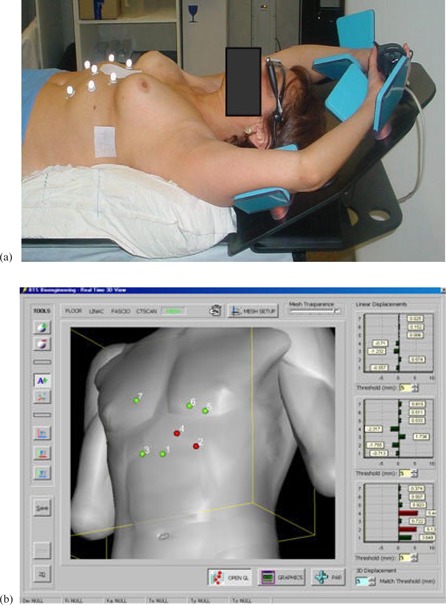
Patient with passive markers and eyewear viewer (a) showing in real‐time marker displacements in LL, CC, and AP directions (with respect to a reference configuration (b)).

Real‐time visual feedback, prompting instantaneous (at ∼30Hz video refresh rate) information of the inspiration level, was obtained through an eyewear viewer (SV‐6 PC Viewer; MicroOptical Co., Westwood, MA) (Fig. 1) connected to the ELITE data logger, showing the optical marker displacements in LL, CC, and AP directions, with respect to a reference configuration, as bars. The patient was first instructed to have a reproducible breathing pattern and then was trained to achieve a reproducible DIBH level, keeping the AP bars below a 3 mm threshold. When the bars exceeded the threshold, their color changed from green to red. A configuration of seven optical markers was used, four positioned in stable points to assure a correct setup in FB and three on upper abdomen in points representative of the DIBH maneuver. Acquisition of the reference 3D marker positions was performed at a comfortable level of DIBH and the duration of the breath‐hold was established.

Each patient underwent a FB CT scan and three consecutive DIBH CT series spanning the region across the tumor (usually 20 slices), acquired with 3 mm thick slices. Each DIBH CT series was acquired during a single breath‐hold of about 16 sec (one slice acquired in 0.8 sec).

**Table 1:** Characteristics of the patients enrolled in the feasibility study and in the treatment protocol

*No*.	*Age*	*Sex*	*Site*	*Subsite*	*Comorbidities*	*Thoracic/Liver Surgery*	*Treatment/Feasibility Study*
1	44	F	Left lung	Upper lobe	Hypertension	No	Feasibility study
2	44	F	Right lung	Lower lobe	Hypertension	No	Feasibility study
3	62	F	Mediastinum	Station R4	Heart disease	Yes	Feasibility study
4	48	M	Left lung	Lower lobe	‐	Yes	Feasibility study
5	50	F	Left lung	Upper lobe	‐	No	Feasibility study
6	63	M	Mediastinum	Station 5	COPD	No	Feasibility study
7	59	M	Left lung	Lower lobe	Heart disease	No	Feasibility study
8	61	M	Right lung	Lower lobe	‐	Yes	Treatment
9	53	F	Right lung	Medium lobe	‐	No	Treatment
10	66	M	Right lung	Medium lobe	Left Pneumothorax	No	Treatment
11	53	F	Left lung	Lingula	‐	No	Treatment
12	62	M	Right lung	Lower lobe	‐	No	Treatment
13	60	M	Right lung	Lower lobe	‐	Yes	Treatment
14	54	M	Right lung	Upper lobe	COPD	Yes	Treatment
15	69	F	Left lung	Upper lobe	Left Diaphragm Relaxation	No	Treatment
16	43	F	Liver	Hilar node	‐	Yes	Feasibility study
17	58	M	Liver	V segment	‐	No	Treatment
18	59	M	Liver	VII segment	‐	Yes	Treatment
19	61	F	Liver	IV segment	‐	No	Treatment
20	59	M	Liver	VII segment	‐	Yes	Treatment
21	68	F	Liver	VII segment	‐	Yes	Treatment
22	56	F	Liver	VII segment	Hypertension	Yes	Treatment
23	54	F	Liver	IV segment	‐	No	Treatment

Moreover, shallow inspiration and expiration breath‐hold CT scans centered on the lesion to assess the extent of tumor motion under FB conditions were also acquired.

All CT scans were registered on the FB CT scan using a pair objects technique provided by the TPS; three landmarks on the patient tray and one vertebral body were used. The accuracy of the image fusion, determined by evaluating the contours of fixed anatomical structures, was below 1 mm. The GTV was contoured on each slice by a single radiation oncologist. We measured an intra‐observer variability of 1.5 mm, evaluated by repeating three times the tumor delineation of two patients and considering the shift in the tumor center of mass.

**Figure 2:**
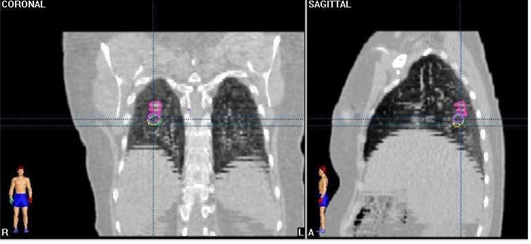
Reproducibility of tumor position during repeated DIBH CT scans fused on the FB CT scan (shown in pink).

In Fig. 2, three DIBH CT series registered on the FB scan are shown for a lung patient enrolled in the feasibility study. Artifacts in tumor volume and position in the FB CT scan are visible in the sagittal and coronal CT reconstructions.

We investigated the correlation between the GTV and the optical markers displacement from FB to DIBH, in terms of entity and reproducibility. Tumor displacement was assessed by evaluating the displacement of the GTV center of mass along the LL, CC, and AP directions between FB and DIBH. As a surrogate of surface motion, we considered the mean value of the positions of abdominal optical markers used to guide the DIBH maneuver. The reproducibility of tumor displacement was evaluated considering the SD of the position of the GTV center of mass between consecutive DIBH CT scans, while the reproducibility of marker displacements was evaluated considering the mean SD of the coordinates of abdominal markers digitized on the TPS.

### C. clinical implementation of DIBH protocol

#### C.1 CT simulation

Each patient underwent a full DIBH CT scan, split into segments lasting about 15 sec each, including all involved organs for the DVH analysis and three consecutive DIBH CT scans, centered on the lesion, to evaluate tumor position reproducibility. A full FB scan for setup and shallow inspiration and expiration breath‐hold scans centered on the lesion to assess the extent of tumor motion were also acquired. The same procedure as described in the feasibility study (Materials and Methods Section B above) was followed.

A verification CT in DIBH centered on the lesion was acquired before each treatment section, since a cone beam CT was not available on the linac.

So far, 15 patients have been treated with the DIBH protocol and their characteristics are shown in Table 1 (Patients #8–15, #17–23).

#### C.2 Treatment planning

The treatment technique consisted of a single or multiple noncoplanar arcs of 6 MV photon beams conformed to the PTV by means of a dynamic micromultileaf collimator (m3, BrainLAB AG). For metastases, the CTV was equal to GTV, while for primary tumors the CTV was defined as the GTV plus 5 mm margin. The PTV was defined by adding to the CTV drawn on the DIBH CT scan the same margins of the FB plan. Patient‐specific margins of 5–8 mm in LL and 8–18 mm in both AP and CC were used, taking into account organ motion and 3 mm isotropic setup margin.^(23)^ An additional margin of 3 mm was added to the PTV to account for beam penumbra. Moreover, an extra 2 mm margin was used to compensate the reduced accuracy of our dose calculation algorithm, not accounting for lateral electronic disequilibrium in the lung.

We compared the CTV to PTV margins, derived from the FB method, with the uncertainty‐based margins calculated taking into account the following uncertainties, combined in quadrature, involved in the process: patient specific tumor position reproducibility in DIBH (1.5 SD), setup margin, intra‐observer variability in tumor delineation, and uncertainty in image fusion.

Treatment doses, prescribed at the isocenter, ranged between 30 Gy delivered in 2 fractions and 54 Gy delivered in 3 fractions with 48 hours interval.

#### C.3 Treatment delivery

Patients were treated on a Varian Clinac 600 C/D (Varian Medical Systems, Palo Alto, CA) equipped with an electronic portal imaging device. Patient setup was performed according to ExacTrac. Following initial manual alignment with skin marks and lasers, the position of the couch was automatically adjusted on the basis of optical marker localization in order to achieve a treatment position where the markers matched the reference configuration defined on the treatment planning CT, especially the stable ones. Final patient setup verification was performed by means of a couple of orthogonal electronic portal images acquired in FB and compared with the corresponding DRRs. Bony structures stable during respiration, such as vertebral spine, were used to verify the correct patient position in FB as determined by ExacTrac. Each irradiation was split in 12–15 consecutive breath‐holds lasting about 15–20 sec each, using high‐dose rate (500 MU/min). During each breath‐hold, the beam was switched on manually when the patient reached the correct level given by the ELITE system and switched off before the operator told the patient to freely breath.

We analyzed the intra‐ and inter‐DIBH reproducibility, considering the mean SD and the root mean square error (RMS) of marker displacements, respectively.

## III. RESULTS

### A. Feasibility study of the reproducibility of DIBH technique

Real‐time visual feedback of marker displacements to the voluntary subjects led to a significantly higher repeatability of surface localization during DIBH, with overall mean 3D displacement of 2.2±0.8mm, compared to a mean value of 3.2±1.1 obtained with verbal instructions given by an operator (p=0.0003).

### B. CTV to PTV margins

In Table 2 are shown for all patients the SD of the GTV positions in repeated DIBH CT scans and the uncertainty‐based CTV to PTV margins calculated combining in quadrature the patient‐specific tumor position reproducibility in DIBH (1.5 SD), the setup margin (3 mm), the intra‐observer variability in tumor delineation (1.5 mm), and the uncertainty in image fusion (0.5 mm).

The mean difference between uncertainty‐based margins in DIBH and the standard ones determined with the FB method (used for the treatment) was −1.9±0.5mm in LL, −5.0±1.5mm in AP, and −6.0±1.8mm in CC directions.

Despite reduced respiratory motion, CTV to PTV margins were not reduced so far, first because we want to acquire more confidence with the technique. The second reason concerns lung treatments and it is related to the dose calculation algorithm implemented in our TPS (see Discussion section below).

All lung patients exhibited a significant decrease in the PTV for the DIBH plan compared to the one obtained in the FB plan, due to target immobilization (p=0.003). We observed an overall mean variation of −13.0%±9.3% with a maximum value of −30.9%±3.1%. For patients with liver lesions, although the tumor was visible in all CT scans allowing us to evaluate the center of mass reproducibility during repeated DIBH CT scans, we did not compare the volumes, since we found some differences depending on the time interval from the contrast medium injection and the CT scan acquisition.

**Table 2:** Reproducibility of the GTV positions in repeated DIBH CT scans and the uncertainty‐based margins calculated on an individual basis

	*SD of GTV Positions in Consecutive DIBHs*			*CTV‐PTV Margins*		
*Patient N°*	*LL (mm)*	*AP (mm)*	*CC (mm)*	*LL (mm)*	*AP (mm)*	*CC (mm)*
1	0.9	0.5	1.6	3.6	3.5	4.2
2	1.0	1.4	2.9	3.7	4.0	5.5
3	0.3	0.8	0.4	3.4	3.6	3.4
4	0.2	1.7	1.8	3.4	4.2	4.3
5	0.4	0.1	1.5	3.4	3.4	4.1
6	0.4	1.7	2.1	3.4	4.2	4.6
7	0.9	0.9	0.8	3.7	3.7	3.6
8	0.7	0.5	1.4	3.6	3.5	4.0
9	1.2	3.6	3.3	3.8	6.4	6.0
10	0.5	0.6	0.5	3.5	3.5	3.5
11	0.1	0.1	1.8	3.4	3.4	4.3
12	0.2	1.1	1.5	3.4	3.8	4.1
13	0.4	0.2	0.5	3.4	3.4	3.5
14	0.2	0.5	0.9	3.4	3.5	3.7
15	0.4	1.0	0.4	3.4	3.7	3.4
16	0.6	0.5	0.4	3.5	3.5	3.4
17	0.9	0.9	1.2	3.6	3.7	3.8
18	1.1	1.6	1.3	3.8	4.1	3.9
19	0.4	0.8	1.3	3.4	3.6	3.9
20	0.0	1.0	1.1	3.4	3.7	3.8
21	1.4	0.5	1.6	4.0	3.5	4.2
22	1.9	1.9	1.7	4.4	4.4	4.2
23	1.6	1.5	1.0	4.2	4.1	3.7
mean	0.7	1.0	1.3	3.6	3.8	4.1
SD	0.5	0.8	0.8	0.3	0.6	0.6

### C. Displacement of target and passive markers between FB and DIBH

Entity and reproducibility of tumor and markers displacement from FB to DIBH for all patients are shown in Table 3.

**Figure 3:**
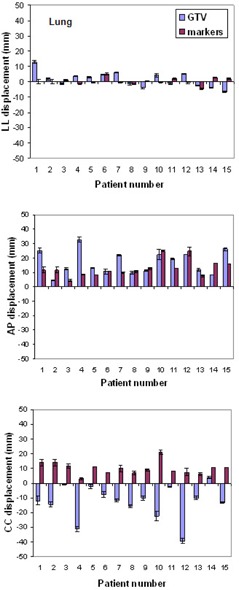
Displacement of GTV and passive markers between FB and DIBH in all directions for lung lesions.

**Figure 4:**
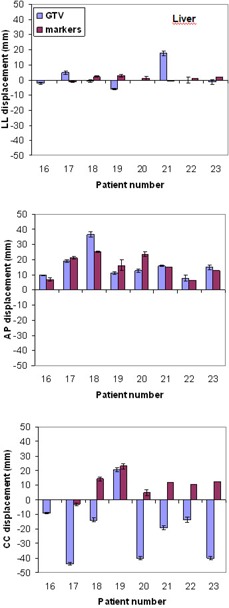
Displacement of GTV and passive markers between FB and DIBH in all directions for liver lesions.

Figures 3 and 4 show the tumor and markers displacement from FB to DIBH along the three directions for patients with lung and liver diseases, respectively. For lung tumors situated in the lower lobes, the largest displacement was observed in the caudal direction with a maximum value up to 40 mm, while tumors located in the upper lobes displayed maximal displacement along the AP direction, with a maximum value of 33 mm. Most tumors showed minimal movement along the LL direction, but for three patients it was larger than 5 mm.

We did not find any correlation (Pearson correlation coefficient) between the marker and the tumor displacement in any directions among the patients.

All patients with liver lesions, except one, exhibited a caudal displacement of the target, caused by the increased lung volume pushing down the liver. The only patient showing target displacement in the opposite direction had a lesion very close to the diaphragm pushed up during DIBH. Relevant displacements were also observed in AP direction. One lesion, located on the diaphragm, exhibited a displacement of 17.7 mm in LL direction.

A correlation between tumor and marker displacement in CC direction (regression line $r^{2}=0.7178,p=0.0021$) has been found for liver lesions.

We observed a high variability of tumor displacement between FB and DIBH among the patients, which may be caused by different respiration modality, thoracic or abdominal, as well as position of the lesion.

Data from the verification CT acquired before each treatment section confirmed the reproducibility of tumor position during DIBH assessed during the simulation CT for all patients.

**Table 3:** Entity and reproducibility of tumor and markers displacement from FB to DIBH CT scans for all patients enrolled in the feasibility study and in the treatment protocol

			*LL*		*AP*		*CC*	
			Mean+SD(mm)	*Range (mm)*	Mean+SD(mm)	*Range (mm)*	Mean+SD(mm)	*Range (mm)*
Lung	Tumor	Displacement	1.4±5.0	−6.6/12.9	16.8±8.1	4.5/33.0	−12.7±11.5	−39.6/3.9
		Reproduc.	0.5±0.3	0.1/1.2	1.0±0.9	0.1/3.6	1.4±0.9	0.4/3.3
Markers	Displacement	0.3±2.2	−4.7/5.2	12.7±5.8	4.4/24.8	10.1±4.2	3.0/21.0
	Reproduc.	0.6±0.5	0.3/1.5	1.1±0.8	0.3/3.0	1.6±0.7	0.8/2.7
Liver	Tumor	Displacement	1.4±7.2	−6.2/17.7	16.0±9.1	7.7/36.7	−19.9±21.4	−43.9/20.5
		Reproduc.	1.0±0.6	0.0/1.9	1.1±0.5	0.5/1.9	1.2±0.4	0.4/1.7
Markers	Displacement	1.0±1.5	−1.4/2.8	15.8±7.1	6.1/25.0	10.6±8.0	−3.0/23.0
Reproduc.	0.7±0.4	0.3/1.1	1.6±1.2	0.6/3.6	1.6±0.4	1.0/2.0

### D. Patient performance during the DIBH treatment

The mean number of breath‐holds per fraction was 10.8±2.1 (range: 6.0 to 16.0). The mean number of frames acquired during a single breath‐hold was 288±97, with mean breath‐hold duration of about 15 sec and a sample rate of 20 Hz. Intra‐ and interbreath‐hold reproducibility, as determined by marker displacements, for all treated patients is shown in Table 4.

We found larger RMS errors in LL and CC directions due to the fact that, although the patients could have real‐time feedback of markers displacements along the three directions, they were told to reproduce as better as possible their reference positions along the AP direction.

**Table 4:** Intra‐ and interbreath‐hold reproducibility of marker displacements for all treated patients

	*LL*		*AP*		*CC*	
*Reproducibility*	Mean+SD(mm)	*Max (mm)*	Mean+SD(mm)	*Max (mm)*	Mean+SD(mm)	*Max (mm)*
Intra ‐ DIBH	0.5±0.5	1.6	0.6±0.5	1.7	0.7±0.7	2.0
Inter ‐ DIBH	2.2±0.9	3.9	1.6±0.4	2.3	2.3±0.7	3.7

## IV. DISCUSSION

### A. Feasibility study on the reproducibility of DIBH technique

Preliminary study showed that self‐visual feedback increased the reproducibility of the patient surface. Our results are in agreement with other studies reported in the literature.^(26)^ Some authors found a reproducibility of tumor position under patient self breath‐hold without any respiratory monitoring device, with a difference of tumor position less than 3 mm in all directions.^(10)^

### B. CTV to PTV margins

The PTV was smaller in DIBH compared to FB, even using the same margins derived from the FB plan, due to the fact that artifacts in shape and location of the lesion caused by the CT acquisition were minimized. Since the tumor is immobilized during DIBH, it might be possible to reduce the internal margin, taking into account only the residual tumor motion during breath‐hold. Nevertheless, a margin reduction should be considered carefully if a pencil beam algorithm is used for dose calculation, as in our TPS. Since DIBH reduces lung density, the effect of a broadened penumbra at field edges due to electronic disequilibrium will be increased and may cause under dosage of targets, as pointed out by Fogliata et al.^(27)^ Hanley et al.^(20)^ showed that for 6 MV photon beams the penumbra broadening should have little clinical significance for DIBH treatments. Yorke et al.^(28)^ found, using Monte Carlo, that lateral disequilibrium caused more decreased target coverage for DIBH than for FB. However, if DIBH enables higher prescription doses exceeding 10%, despite lateral disequilibrium, higher doses would be delivered to target volume.

We have planned to change our dose calculation algorithm with a more accurate one. In the meantime, the implementation of the DIBH technique allowed us to escalate the dose for selected patients. Two patients with a primary lung tumor received a treatment dose of 54 Gy in 3 fractions, while our standard protocol was 45 Gy in 3 fractions.

### C. Displacement of target and passive markers between FB and DIBH

The tumor displacement between FB and DIBH varied significantly among patients. We could not find any correlation between the movement of the markers used to guide the DIBH and tumor displacements for patients with lung lesions, while a weak linear regression was found in CC direction for patients with liver metastases. Nevertheless, this is not an important issue once the tumor position is reproducible with regard of the external markers position.

For each patient it is important to ensure that the tumor position is reproducible with respect to the external markers configuration by acquiring repeated DIBH CT scans during simulation. The availably of a cone‐beam CT (CBCT) on the linac would allow one to acquire a DIBH‐gated CBCT in order to verify the tumor reproducibly just before the treatment.

Our results are in agreement with those obtained by other investigators.^20^, ^29^

### D. Patient performance during the DIBH treatment

Only patients who could achieve sufficient reproducibility of DIBH after the training were included in the treatment protocol. All patients were able to complete the stereotactic treatment, some of them exhibiting fatigue at the end of the fraction.

Berson et al.^(30)^ found a reduction of treatment time by a factor of 2 using breath‐hold compared to gating. This is particularly important for hypofractionated stereotactic treatments, where high doses per fraction are delivered.

## V. CONCLUSIONS

The DIBH technique, supported by an opto‐electronic system, provides a simple and noninvasive method to minimize breathing motion for collaborative patients. A visual feedback provided to the patient led to a higher reproducibility of the technique. We strongly recommend an accurate CT study for each patient to evaluate the reproducibility of tumor position during the DIBH maneuver with respect to the external markers configuration.
